# Scaling Up Citizen Workshops in Public Libraries to Disseminate and Discuss Primary Care Research Results: Quasi-Experimental Study

**DOI:** 10.2196/39016

**Published:** 2022-08-19

**Authors:** José Massougbodji, Hervé Tchala Vignon Zomahoun, Evehouenou Lionel Adisso, Jasmine Sawadogo, Valérie Borde, Cynthia Cameron, Hélène Moisan, Jean-Sébastien Paquette, Zamzam Akbaraly, Lëa-Kim Châteauneuf, Geneviève David, Bibliothèques2 Group, France Légaré

**Affiliations:** 1 Department of Social and Preventive Medicine Laval University Québec, QC Canada; 2 First Nations of Quebec and Labrador Health and Social Services Commission Québec, QC Canada; 3 Centre d’excellence sur le Dialogue entre les scientifiques et le public Québec, QC Canada; 4 Department of Family Medicine and Emergency Medicine Laval University Québec, QC Canada; 5 Bibliothèque de Québec Québec, QC Canada; 6 Patient and Public Partnership Research Strategy Component Quebec SPOR-SUPPORT Unit Laval University Québec, QC Canada; 7 Direction des bibliothèques Service de la culture - Ville de Montréal Montreal, QC Canada; 8 Centre d'excellence sur le partenariat avec les patients et le public Centre de Recherche du Centre Hospitalier de l'Université de Montréal Montreal, QC Canada; 9 See Acknowledgments Québec, QC Canada; 10 VITAM – Centre de recherche en santé durable Centre intégré universitaire de santé et services sociaux de la Capitale-Nationale Québec, QC Canada

**Keywords:** scaling up, knowledge translation, dissemination strategies, integrated knowledge translation, public libraries, citizen workshops, potentially inappropriate medicines

## Abstract

**Background:**

Little is known about engaging patients and stakeholders in the process of scaling up effective knowledge translation interventions targeting the public.

**Objective:**

Using an integrated knowledge translation approach, we aimed to scale up and evaluate an effective pilot program to disseminate research results in public libraries.

**Methods:**

We conducted a scaling-up study targeting the public. On the basis of our successful pilot project, we codeveloped and implemented a large-scale program of free citizen workshops in public libraries, in a close research partnership with stakeholders and patient representatives. Citizen workshops, each facilitated by 1 participating physician and 1 science communicator, consisted of a 45-minute computer-assisted presentation and a 45-minute open exchange. The intervention outcome was knowledge gained. The scale-up outcomes were satisfaction, appropriateness, coverage, and costs. An evaluation questionnaire was used to collect data of interest. Both quantitative and qualitative analyses were performed.

**Results:**

The workshop theme chosen by the patient and stakeholder representatives was the high prevalence of medication overuse among people aged ≥65 years. From April to May 2019, 26 workshops were conducted in 25 public libraries reaching 362 people. The mean age of participants was 64.8 (SD 12.5) years. In total, 18 participating physicians and 6 science communicators facilitated the workshops. Participants reported significant knowledge gain (mean difference 2.1, 95% CI 2.0-2.2; *P*<.001). The median score for overall public satisfaction was 9 out of 10 (IQR 8-10). The public participants globally rated the workshops as having a high level of appropriateness. Coverage was 92% (25/27) of the total number of public libraries targeted. Costs were CAD $6051.84 (US $4519.69) for workshop design and CAD $22,935.41 (US $17,128.85) for scaling them up.

**Conclusions:**

This project successfully established a large-scale and successful implementation science or knowledge translation bridge among researchers, clinicians, and citizens via public libraries. This study provides a model for a dissemination practice that benefits the public by both engaging them in the dissemination process and targeting them directly.

## Introduction

### Scale Up of Health Interventions

Much research remains on the shelf. The average delay for integrating research findings into healthcare delivery is still estimated in units of decades, despite recent advances made in implementation science or knowledge translation (both hereafter referred to as KT) [1-3]. While KT attempts to address this gap, most KT interventions target health professionals and ignore the public [4]. Within the clinical context of primary care, the public and patients are key end users of research findings. They should be informed about new evidence that could benefit them and be involved in any KT process that targets them [5,6]. Without patient and stakeholder involvement in judging the relevance of the knowledge being transferred, the new knowledge may not be patient-centered and remains in the hands of the professionals delivering care. Any patient engagement in KT is still mostly *low level* engagement [7]. Most health intervention pilot projects, even if proven effective, remain on the shelf. One way to bring effective pilot projects off the shelf is to scale them up so that their benefits reach a broader population. Scaling up is becoming an important motor of KT and is developing into a science unto itself [8,9]. The process of *scaling up* can be defined as “deliberate efforts to increase the impact of successfully tested health innovations so as to benefit more people and to foster policy and program development on a lasting basis.” [10].

KT interventions rarely target the public directly, who are their potential if not actual patients. Even web-based surveys are unrepresentative of the public, as they only reach people with educational and technological resources [11]. Meanwhile, public libraries are known for their extensive population reach, as they attract homeless and other marginalized patrons [12-14]. Their patrons also see public libraries as a valuable resource for medical information [15,16]. Furthermore, unlike other service-providing institutions (eg, medical and some social welfare institutions), libraries are widely trusted by the public [14]. Therefore, they can be an excellent avenue for disseminating accurate medical information to users. Ultimately, this could lead to increased public expectations and demands for care that is more patient-centered, thus changing the dynamics of care between patients and providers [17] by fostering positive behaviors such as shared decision-making by both partners in the care relationship [18].

### Workshops in Public Libraries as Effective KT Interventions

In 2017, we established a proof of concept on the dissemination of research results to the public through workshops in public libraries. These pilot workshops, designed to raise awareness of new knowledge in primary care research, were conducted across 9 public libraries in Quebec City, Quebec, Canada. We demonstrated evidence of their effectiveness by measuring the acquisition of knowledge among participants [19]. First, we hypothesized that one of the reasons for our pilot’s positive results was the library setting. Public libraries are free community-based civic institutions associated with increasing knowledge at one’s own pace and in accordance with one’s interests. This setting thus helped reduce the usual power differential between health professionals and patients, as these potential patients had freely chosen to be present instead of being obliged to hear messages from health professionals. Second, we give credence to our communication strategy, whereby research findings were delivered by physicians who were credible messengers and by a science communicator using plain language accessible to lay people. Third, we successfully mobilized several key stakeholders, such as physicians, a science communicator, and a public library manager, and attracted the public. Overall, our successful pilot workshops appeared to be an appropriate candidate for scaling up, according to a World Health Organization guide to scale up [10]. Evidence of their effectiveness was sound, observable, and documented. They had already been tested in a setting similar to the target setting. We succeeded in maintaining comparable participation rates for workshops across public libraries, which was a good indication of the generalizability of our project. The model was easily transferable, it matched the values of the target institutions (such as libraries), and similar logistics could be applied. On the basis of our body of evidence, our next step was to investigate how these results would hold on a larger scale; that is, by targeting more public libraries and delivering more workshops. Ultimately, we expected that reaching a larger public and increasing their knowledge would greatly impact population health.

However, there is no point in scaling up KT intervention projects that are not relevant to their target populations. Knowledge must also be accessible to end users. To this end, the integrated knowledge translation (IKT) approach has been increasingly adopted in implementation studies [20]. IKT aims to gather the views of all stakeholders, including knowledge users, throughout the research process in an inclusive, engaging, and interactive manner [21]. It is based on research partnership, the equitable sharing of power, and mutual respect among all stakeholders. The benefits of this approach have been widely demonstrated in the literature [22]. In this scaled-up version of our pilot, we planned to engage patients and stakeholders from start to finish, involving men and women at a *high level* of engagement [23]; that is, in choosing the theme, defining its content, and evaluating its outcomes while maintaining or improving workshop effectiveness.

Therefore, we aimed to scale up an effective pilot program to disseminate research results to the public through citizen workshops in public libraries using an IKT approach, while maintaining fidelity and with equal or improved effectiveness.

## Methods

As no specific reporting guidelines for scaling up studies are available, we used an adapted version of the Standards for Reporting Implementation Studies guidelines to report our study [24]. We also relied on the Template for Intervention Description and Replication reporting guidelines for the description of the intervention [25].

### Study Design

As with the pilot project, we conducted a pre-post intervention study. Participants self-reported both preintervention and postintervention outcome measures only after the intervention was completed (reducing response shift bias for the outcome measures and the burden on participants) [26,27]. Using an IKT approach, we adapted the pilot methodology to engage patients and stakeholders throughout this scaling up study. Therefore, this study was not registered.

### Context

While the pilot project took place in Quebec City, Quebec, Canada, the scaled-up intervention was extended to Montreal, Quebec, Canada, which, similar to Quebec City, is largely Francophone, so the culture and language were similar. However, it should be noted that Montreal has a higher immigrant population and includes the city of Westmount, Quebec, Canada, which is more Anglophone. There are also economic differences between localities, with Westmount being richer than most districts of Montreal and Quebec City.

### Targeted Sites and Population

Convenience sampling was conducted to select libraries in Quebec City, Montreal, and Westmount, which were able to include a citizen workshop in their spring 2019 agenda and had the necessary amenities (ie, video projector, laptop, speaker, and room for 30 people). The target population for our study was public library patrons aged ≥18 years. Their participation was voluntary. To maximize the number of participants in the libraries, and also to ensure a variety of profiles (eg, sex, age, and education level) among all participants, libraries were free to schedule citizen workshops on the dates and times they deemed most convenient (ie, during the workday or in the evenings and weekdays or weekends).

### Planning to Scale Up the Intervention

#### Establishing a Committee

This scale-up study began with the formation of a preliminary project steering committee and was informed by the Canadian Institutes of Health Research Integrated and End-of-grant knowledge translation frameworks [28]. The proposal for scaling up the pilot intervention, that is, a presentation with layman- and user-friendly content followed by an exchange period, was consensually retained by the committee.

#### Name

We named this intervention *citizen workshops* because of the strong involvement and responsibility of all stakeholders in the process: primary care researchers would produce results and make them available to physicians, science communicators, and patient and research partners for dissemination to the public; the public would identify the most relevant results; public libraries would host the workshops; and science communicators would facilitate them.

#### Recruitment

Through a convenience selection, we recruited stakeholder representatives, including 4 experts in patient-oriented research, 1 science communicator, 1 primary care physician, and 2 public library officials. The primary health care researcher, whose results were selected for dissemination, and a patient expert (ie, a patient or informal caregiver trained in research), who was a caregiver of a patient facing the health problem addressed, joined the committee for the remaining stages. The library officials on the committee arranged for invitations to be sent to all public libraries in Montreal, Westmount, and Quebec City and then helped to identify the libraries that would host the citizen workshops. In addition to the libraries' usual information channels (programming pamphlets, websites, and social media platforms), posters, a dedicated website [29] and radio advertisements, social media platforms (Facebook), and newsletters were used to reach the participants. These means of promotion were designed and approved by all final committee members including the patient expert.

The committee decided that each citizen workshop would be moderated by a team consisting of a family physician as the speaker and a science communicator in charge of facilitating and articulating the message in plain language. Thus, researchers and physicians on the committee issued a letter to be included in primary care professional and research organization newsletters, inviting any willing primary care physician (emergency and family medicine) or resident to participate in the project. The only prerequisite was that they had to have good knowledge and practical experience of the health problem addressed. Science communicators were selected by the science communicator member of the steering committee according to their ability to communicate orally in plain language, to lead a constructive discussion with an audience, and to manage the unforeseen (inappropriate questions, speaker forgetting important details or explaining key concepts poorly, or technical or operational mishaps) and their respectful and empathetic attitude.

#### IKT Strategies

We have involved different stakeholders as research partners at all steps of our research process, except the patient caregiver and the representative of winning researchers who were identified and involved after the selection of the research results to be disseminated. The research results selected allowed us to determine the health problem addressed and therefore the relevant profile of patients to be involved as research partners in our study. The relevant patient profile we determined required the permanent support of a caregiver. Therefore, we included as the research partner a caregiver having substantial life experience with the patient having a health problem of interest.

As research partners, the stakeholders contributed to the research process using the following strategies: (1) the members of the preliminary steering committee discussed and approved the aim of the citizen workshops, which was to inform and raise awareness of the research results that would be selected. (2) Then, they identified the theme for which research results would be disseminated in the citizen workshops: a call for research results was issued to all primary health care research teams in the province with a letter, validated by all committee members, encouraging them to submit their research results for free dissemination. The physicians and researchers on the committee helped to identify the best means for disseminating the call for research results throughout the province of Quebec (dissemination networks of primary care research centers, university hospital centers, faculties of medicine, pharmacy, and nursing care in universities in Quebec). (3) Applications were evaluated exclusively by the preliminary committee members who reflected the voice of the main stakeholders, including librarians, primary care physicians, experts in patient-oriented research, and science communicators. (4) All final committee members, including the patient expert and the owner of the selected research results, participated in writing and approving the script for the workshops. (5) Patients and stakeholders on the committee were involved in all stages of the implementation, including workshop observation, data collection, and outcomes evaluation. (6) Meetings among all actors, including the patient expert, were organized on a bimonthly basis. A progress report was sent every 2 months over 18 months. (7) Patients and stakeholders on the committee were assigned to observe all workshops. Their role was to distribute and collect evaluation questionnaires completed by the participants of the citizen workshop. They ensured the smooth running of the workshop and counted the number of participants at the beginning of each citizen workshop. (8) Preliminary results of the data analysis for the project were discussed with stakeholders, and their comments and suggestions were considered in the final interpretation of the results.

### Implementing the Scaled-up Intervention

#### Preparation

To ensure consistency of citizen workshops across libraries, materials for moderation (ie, PowerPoint [Microsoft Inc] presentation, handouts, and notes for each individual moderator) were sent 6 weeks earlier to all participating moderators. They had 2 weeks to familiarize themselves with the materials. Then, two 1-hour preparatory meetings, spaced 2 weeks apart, were held by the committee and all participating moderators. During these meetings, the committee gathered moderators’ feedback on the documents for consideration and sought their approval.

#### Workshop Content

Each citizen workshop was divided into two 45-minute equivalent parts: the first was a computer-assisted presentation of the results, and the second was a knowledge exchange between participants and physician presenters moderated by a science communicator. The knowledge exchange included not only the question-and-answer round but also knowledge sharing through the lenses of scientific evidence, beliefs, personal experiences, and values disseminated about the topic. First, an introductory part raised public awareness of the health issues related to the results by defining terms and providing context. Then followed the actual results of the selected study and a detailed description of their direct impact on the public and potential repercussions on their health. In the knowledge exchange session, the science communicator ensured that any questions from the participants did not seek a personalized medical consultation. This format was identical to that of the pilot phase workshops, except for one major adaptation: the addition of a video clip to the presentation in the first part of the workshop with the testimony of the patient expert associated with the project. We made this change because, in the pilot study, the workshops generated greater gains in knowledge among young people than among older people. We hypothesized that older people might need information presented in a different format to reach them better. On completion of the workshops, participants were left with a handout outlining the research results along with additional documents and resources about the health problem addressed. Detailed information on the content of the intervention and handouts can be available upon request.

#### Maintaining Fidelity

Except for the addition of the video clip to the presentation, efforts were made to maintain fidelity to the piloted workshop concept and content. Workshops were given in French in all libraries, even in areas that were predominantly Anglophone. The same content was offered with moderators having comparable profiles. To maintain fidelity, we had to add some elements to the new contexts; for example, some public libraries did not have projectors for the slide presentations with sound, so we purchased our projection materials.

### Evaluation

#### Outcomes of Scaling Up

Outcomes of scaling up were related to selected aspects of acceptability and appropriateness of citizen workshops among participants, workshop coverage, time, and costs.

According to the taxonomy of implementation outcomes by Proctor et al [30], acceptability is the perception among stakeholders that an innovation is agreeable or satisfactory while appropriateness is the perceived fit, relevance, or compatibility of the innovation. These outcomes were measured using 12 closed-ended questions regarding participants’ opinions of the workshop. Acceptability was measured using 3 questions that focused on the structure of the activity, 3 questions on workshop facilitation, and 2 questions on whether the workshop met their expectations and whether they would recommend it to others. Participants also indicated their overall satisfaction with the workshop using a discrete 11-point scale where 0 corresponded to unsatisfied and 10 corresponded to fully satisfied.

Appropriateness, on the other hand, was measured using 4 questions on the workshop quality and relevance. Answers to all questions except general satisfaction were chosen from a 4-point Likert scale (ranging from 1, “totally disagree,” to 4, “totally agree”). Qualitative data on participants’ acceptability were also collected from open-ended questions in the evaluation form.

Coverage was determined by determining the ratio between the numerator (ie, the number of libraries that hosted the workshops) and the denominator (ie, the number of libraries targeted for participation).

A partial economic evaluation focusing solely on costs was conducted separately for the workshop design costs and the scaling-up costs to distinguish between modifiable costs related to the scale-up strategy and nonmodifiable costs related to the intervention. Costs for scaling up included remuneration of steering committee members, medical moderators, science communicators, and patient observers; purchase of the necessary equipment; and actual delivery of workshops. Expenses related to designing the citizen workshops included fees for steering committee members for designing and writing the workshop script, as well as filming the video clip incorporated into the presentation.

#### Intervention Outcomes

The main outcome of the intervention was knowledge gain, as perceived by the participants about the health problem addressed. To assess this, we adapted the self-administered questionnaire used in our pilot study [19]. This questionnaire was administered to participants at the beginning of the workshop, and they were invited to complete it at the end (Multimedia Appendix 1). They rated their knowledge using a discrete scale from 0 (very low) to 10 (very high).

Data were also collected on participants’ sociodemographic characteristics, such as age, sex, and highest level of education. Finally, other variables pertaining to workshop characteristics were collected by direct observation during their delivery: the time of day during which the workshops were held, the presence or absence of the patient expert as an observer during the workshop, and whether the speaker was a physician or resident.

#### Analysis

First, we performed a descriptive analysis of the participants according to their sociodemographic characteristics and the workshops they attended, their opinions, and their levels of satisfaction and knowledge.

We used a 2-tailed paired *t* test to compare self-reported pre- and postknowledge levels [31,32]. Comparative analyses of the knowledge gain were then conducted according to the characteristics of not only the participants but also the workshop in which they participated. To this end, univariate linear regression models of knowledge gain were constructed [33]. To assess how knowledge gains would vary across public libraries, comparisons were also made according to the workshops’ moderators (ie, each facilitator, speaker, and pair of moderators) using an ANOVA test [34]. However, given the skewed distributions of knowledge levels and gain, sensitivity analyses were performed: first, the Wilcoxon signed rank comparison test was used to compare before and after median knowledge levels [35]. Second, unmatched rank tests on the median and nonparametric multiple comparisons were performed using the SAS NPAR1WAY procedure [36]. Statistical significance was defined as *P*<.05 (2-sided test).

All analyses were performed in the SAS software (version 9.4; SAS Institute Inc). Qualitative data collected through open-ended questions were transcribed by 1 author (JS) and analyzed using an iterative deductive method discussed with team members. For the economic evaluation, we calculated the sum of expenses separately for the scaling-up strategies and for the design of citizen workshops. The cost results are presented in Canadian dollars. On April 5, 2019, CAD $1 was equal to US $0.75.

### Ethics Approval and Consent to Participate

Ethics approval was granted from the *Comité d’éthique du Centre intégré universitaire de santé et services sociaux de la Capitale-Nationale* under project 2019-1513. Informed consent was first obtained verbally from the study participants at the beginning of each conference as the conferences were recorded. Written consent was obtained from the participants who agreed to complete the conference evaluation forms. The study protocol was approved by the ethics committee.

## Results

### Overview

Following the committee’s call for research results, 5 research teams submitted their results. The results selected that responded to public or patient interest, according to the selection committee, addressed the high prevalence of the use of potentially inappropriate medicines among people aged ≥65 years in Quebec [37].

### Population

A total of 25 libraries, including 9 in Quebec City and 16 in Montreal, agreed to host the citizen workshops. From April 4 to May 29, 2019, 26 workshops were offered in Montreal, including 1 workshop in Westmount and 10 workshops in Quebec City, with 1 library agreeing to host 2 workshops. A total of 18 physicians were mobilized to present the selected findings, and 6 facilitators were recruited. Consequently, 22 distinct pairs of moderators were assembled.

**Figure 1:**
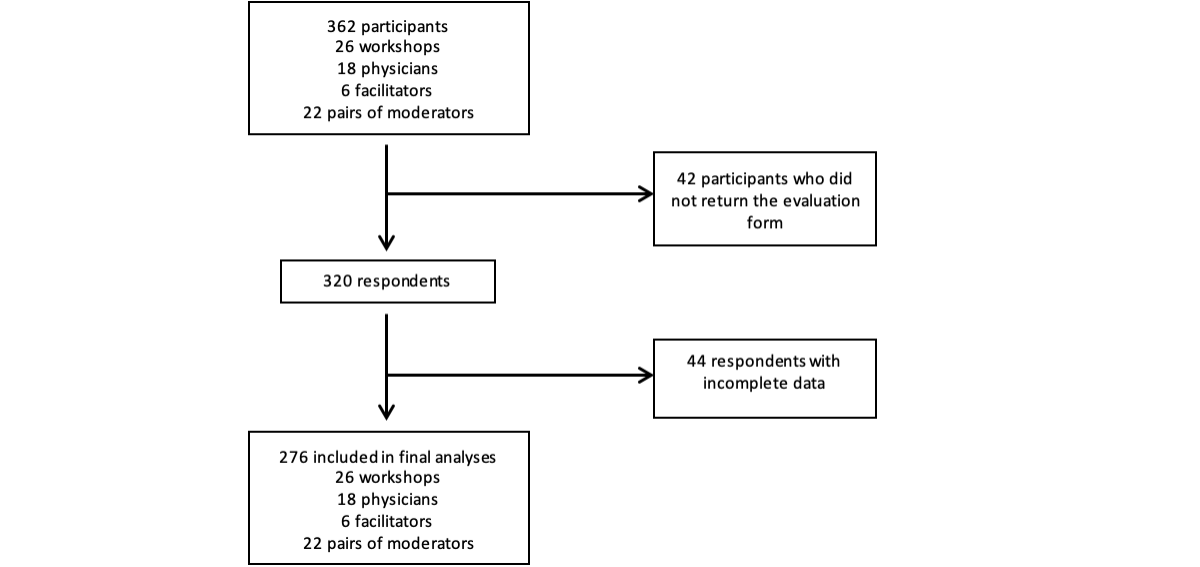
A flowchart of participants in citizen workshops.

The citizen workshops drew 362 participants, with a mean of 13.9 (SD 6.0) participants per workshop. The evaluation questionnaire was returned by 320 participants (Figure 1). Table 1 presents the sociodemographic characteristics of the participants and characteristics of the workshops in which they participated. The mean age of the participants from the public was 64.8 (SD 12.5) years. Women accounted for 71.6% (229/320) of the participants from the public, and half had a university-level education (172/320, 53.8%). Approximately half (150/320, 46.9%) attended workshops in the evening, and 18.1% (58/320) had a patient partner present at their workshop. Most participants (279/320, 87.2%) had a physician as the speaker.

**Table 1:** Distribution of participants and citizen workshops and their characteristics.

			Montreal and Westmount (n=178)	Quebec (n=142)	Total (N=320)
**Participant characteristics**					
	**Sex, n (%)**				
		Female	128 (71.9)	101 (71.1)	229 (71.6)
		Male	38 (21.4)	38 (26.8)	76 (23.8)
		Missing data	12 (6.7)	3 (2.1)	15 (4.7)
	**Highest educational level, n (%)**				
		Secondary or lower	29 (16.3)	25 (17.6)	54 (16.8)
		College	42 (23.6)	34 (23.9)	76 (23.8)
		University	94 (52.8)	78 (54.9)	172 (53.8)
		Missing data	13 (7.3)	5 (3.5)	18 (5.6)
	**Age (years)**				
		Value, mean (SD)	65.5 (12.4)	64 (12.6)	64.8 (12.5)
		Missing data (participants)	13	5	18
**Workshop characteristics**					
	**Time of day, n (%)**				
		Morning	34 (19.1)	47 (33.1)	81 (25.3)
		Afternoon	81 (45.5)	8 (5.6)	89 (27.8)
		Evening	63 (35.4)	87 (61.3)	150 (46.9)
	**Presence of the patient partner, n (%)**				
		Present	58 (32.6)	0 (0)	58 (18.1)
		Absent	120 (67.4)	142 (100)	262 (81.9)
	**Qualification of physician speaker, n (%)**				
		Physician only	163 (91.6)	116 (81.7)	279 (87.2)
		Physician + resident	0 (0)	20 (14.1)	20 (6.6)
		Resident only	15 (8.4)	6 (4.2)	21 (6.6)

### Outcomes

#### Outcomes of Scaling Up

##### Coverage

Of the 27 public libraries initially planned for the citizen workshops, 25 held workshops, corresponding to a coverage of 92%.

##### Acceptability and Appropriateness of Citizen Workshops, According to the Public

**Figure 2:**
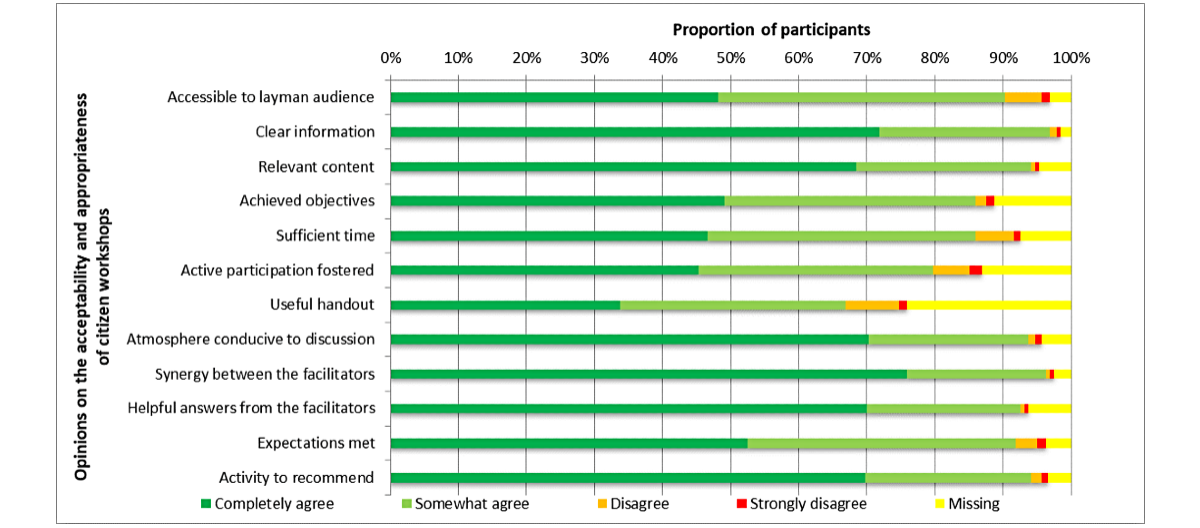
Public participants’ opinions on citizen workshops (N=320).

The median level of overall satisfaction was 9 (IQR 8.0-10) out of 10. With regard to qualitative data, participants pointed out the good quality of the PowerPoint presentation. They particularly liked the inclusion of the interview with the patient partner in the layout of the presentation. This could be considered an indicator of the value of the patient caregiver in IKT. Many participants also perceived and praised the effort to communicate the research results in plain language through the PowerPoint presentation and during workshop facilitation. However, participants expressed some negative impressions, notably that several libraries were open-plan concept and therefore did not have dedicated rooms for this type of activity. Although most participants found the length of the workshops adequate (275/320, 86%), some found that there was not enough time to discuss their concerns. The lowest approval score was obtained for an item that assessed whether their active participation had been encouraged (255/320, 79.7%). However, for the same item, a high rate of missing responses (42/320, 13.1%) was noted. Regarding the moderation of the workshops, most participants reported that the moderators provided an atmosphere conducive to discussion (299/320, 93.5%) and gave them useful answers (296/320, 92.5%). They also appreciated the enthusiasm of the moderators and their complementarity (308/320, 96.2%). Finally, most participants felt that the workshop met their expectations (294/320, 91.9%), and 94.1% (301/320) recommended the activity to others (Figure 2).

In terms of appropriateness, more than 9 out of 10 participants found that the citizen workshops were accessible to a layman audience and that the information presented to them was clear and relevant. However, a low agreement was obtained regarding the usefulness of the documentation provided to them (214/320, 66.9%). This was also the item for which the proportion of missing responses was the highest (79/320, 24.8%). However, many participants found that information in the handouts was too brief, and 1 participant suggested a more substantial document with more information such as examples, useful websites, and a detailed outline of the presentation.

##### Cost and Time

###### Workshop Design

In total, 16 people were mobilized to participate in the committee. Regarding the design of citizen workshops, costs were mainly the fees of the science communicator member of the steering committee for the writing of the workshop’s script and for the shooting of video clips embedded in the presentation. These costs were CAD $6051.84 (US $4519.69). The patient caregiver who had worked for the Quebec Strategy for Patient-Oriented Research Support for People and Patient-Oriented Research and Trials Unit did not receive additional compensation in our study context. The script revision and video clip editing were free, as they were performed by other members of the steering committee with the tools already at their disposal in their workplaces.

###### Scaled-up Workshop Delivery

None of the steering committee members billed for their time since they were professionals who were already paid in their respective workplaces, except the science communicator and the patient expert. Their fee for scale up was CAD $3511.05 (US $2622.15). A software was purchased for the posters and the website creation at a cost of CAD $453.10 (US $338.39). The preparatory meetings for the scaled-up workshops, in terms of travel, per diem, and food, cost CAD $4380.12 (US $3271.20). For the scaled-up delivery of the citizen workshops, 7 external observers were mobilized in addition to the 24 moderators (18 physicians and 6 facilitators). The external observers were research assistants and graduate students. They were mandated to give and collect the evaluation questionnaire completed by participants. They also counted the number of participants and noted any incident occurred during the citizen workshop. The per diem, travel, and accommodation expenses of observers and moderators totaled CAD $13,620.65 (US $10,172.31). The material used during the workshops (office supplies, recorders, and pointers) was evaluated at CAD $970.49 (US $724.79). Total costs for scaling up the intervention were CAD $22,935.41 (US $17,128.85). Therefore, the overall cost for the project was CAD $28,987.25 (US $21,648.55).

The duration of the scaling-up process using the IKT approach, from the creation of the steering committee to the beginning of the citizen workshops, was 17 months and 8 months, respectively, longer than that of the pilot project.

#### Intervention Outcomes

##### Knowledge Gain

The final analyses were carried out on 276 participants after removing those whose information on their level of knowledge about potentially inappropriate medicines either before or after the citizen workshops was missing. On a knowledge scale of 0 to 10, participants reported that they were, on average, fairly well informed about MIPs before the citizen workshops (mean 6.2, SD 1.8) and more so afterwards (8.2, SD 1.4). This represented a significant (*P*<.001) mean increase in knowledge of 2.1 (95% CI 2.0-2.2). Neither the range of participants’ sociodemographic profiles, the workshop characteristics, nor the variety of workshop moderators (as individuals or as pairs) appeared to modify the effect of the workshop on knowledge gain (Table 2).

These results were confirmed in our sensitivity analysis (Multimedia Appendix 2).

**Table 2:** Comparison of knowledge gain among participants in citizen workshops (N=276)^a^.

Characteristics				Population, n		*β* (95% CI)		*P* value^b^
**Participant characteristics**								
	**Sex**							.97
		Male (reference female)	208		.01 (−0.46 to 0.48)			
	Age (years)		276		−.02 (−0.02 to 0.00)		.06	
	**Highest educational level**							.28
		Up to secondary (reference university)	48		.01 (−0.54 to 0.56)			
		College (reference university)	70		.37 (−0.10 to 0.85)			
**Workshop characteristics**								
	**Time of day**							.59
		Morning (reference evening)	72		−0.18 (−0.68 to 0.31)			
		Afternoon (reference evening)	76		.10 (−0.39 to 0.58)			
	**Presence of the patient partner**							.38
		Present (reference absent)	51		.23 (−0.29 to 0.75)			
	**Qualification of physician speaker**							.22
		Physician + resident (reference physician only)	18		−0.72 (−1.54 to 0.10)			
		Resident only (reference physician only)	19		.05 (−0.75 to 0.84)			
	According to the physician speaker		18^c^		N/A^d^		.63^e^	
	According to the facilitator		6^f^		N/A		.47^e^	
	According to the pair of moderators		22^g^		N/A		.60^e^	

^a^N=276 (after deletion of observations with missing variables).

^b^*P* value of linear bivariate regression.

^c^Number of physician’s groups.

^d^N/A: not applicable.

^e^*P* value of ANOVA test.

^f^Number of facilitator’s groups.

^g^Number of pair of moderator’s groups.

##### Harms

No harm was reported from stakeholders or workshop participants.

## Discussion

### Principal Findings

We aimed to evaluate the scaling up of an effective pilot program to disseminate research results through citizen workshops in public libraries. The main departure of the scaled-up intervention from strict fidelity to the pilot intervention was that we adopted an IKT approach to ensure that the citizen workshops faithfully reflected the needs and interests of patients and other stakeholders at every step of the intervention. We achieved high coverage of the project to scale up the workshops, which generated high levels of satisfaction among participants and high levels of acceptability and appropriateness. Participants in the scaled-up citizen workshops also reported an increase in their knowledge level of the subject being discussed. These findings lead us to make the following observations.

First, we achieved high coverage for the scaling-up citizen workshop. This finding could be explained, in part, by the topic being disseminated that was of great interest to most public library users (old people). Another explanation could be that the citizen workshop was integrated into the conference program of participating public libraries. Therefore, there was no additional logistical management that could limit the participation of public libraries.

Second, our scaled-up citizen workshops led to an increase in knowledge among participants. Interactive workshops have been established as ideal for sharing knowledge across professional and sectoral boundaries [38]. In this project, the interactive aspect was emphasized as much as possible by adding a video clip to the initial format of the workshops to better communicate the patient’s perspective. Although the participants in the *scaling-up* audience were much older than those in the pilot project audience, our scaled-up citizen workshops, in addition to being highly satisfying, led to an improvement in knowledge among these participants. These results confirm the importance of designing a more detailed and inclusive format for citizens’ workshops, regardless of the topic under discussion, to increase knowledge among all age ranges within the audience. However, it should also be noted that these results did not allow us to assess the extent to which an increase in knowledge among public participants produced behavioral changes. A study in the United States that evaluated the midterm impact of after-school nutrition workshops in a public library setting and that targeted adolescents and their parents, a program deemed by the authors to be of low intensity even though it consisted of 5 workshops, did not produce any lasting behavioral change after just 3 months [39]. Our citizen workshops, which were one-time events, sought primarily to raise awareness, with behavioral change as an indirect goal. The next step would be to evaluate the immediate and midterm impacts of citizen workshops among the public by assessing health outcome data related to the themes, both at the time of the workshops and at intervals afterward.

Third, adopting an IKT approach improved our scaling-up results in the following ways: (1) the involvement of library network stakeholders in identifying participating libraries could explain the high coverage of our scaling-up project; (2) prioritizing the public’s perspective to identify the results to be disseminated, adopting a co-constructive approach to designing the workshops, and holding preparatory meetings to allow workshop moderators to make the content of the message their own are all reasons that could explain our positive results in terms of acceptability and appropriateness among the public. These positive findings are also consistent with those of our (non-IKT) pilot project. However, interestingly, they also turned out to be of equal magnitude [19] despite the differences between the pilot project and the scaling-up project. This last observation also holds true for the increase in knowledge. This maintenance of improved outcomes despite the change in subject matter, the involvement of various workshop moderators, and the sociodemographic and linguistic differences within the participating public libraries is likely due to the modification of the intervention by incorporating an IKT approach from start to finish.

Fourth, to the best of our knowledge, this is the first scaling-up study to address such high levels of patient and other stakeholder engagement. Our scaled-up version of the workshop achieved fidelity in terms of being true to the concept and content from one site to another and largely true to the concept implemented in the pilot trial, with the addition of a patient-designed video clip. In this video clip, a caregiver having substantial life experience with the patient told the patient’s story. We involved this caregiver in the rest of the research process once the research results to be disseminated were selected. Therefore, the caregiver contributed as a research partner to the research team meetings; workshop content development, planning, and scaling up; and the revision of different research documents (eg, materials related to citizen workshop). However, our pilot project did not use an IKT approach; thus, in theory, our scaled-up version of the intervention did not meet the strictest fidelity requirements of adhering to the intervention, as outlined in the original pilot design. This raises an interesting question about KT. If new knowledge emerges between the pilot program and the scaling-up phase (eg, evidence about the importance of high-level patient engagement), should the scaled-up intervention maintain fidelity at all costs or should this new knowledge be integrated into the scaled-up version? The science of scaling up must not restrict researchers to reproduce interventions at a scale that excludes important new knowledge. Indeed, we propose that, going forward, the IKT approach should be, as far as possible, an essential and integral dimension of scaling up. At first glance, IKT appears to be a cumbersome approach because it requires constant consultation and adaptation that could slow the process of scaling up [40,41]. However, it ensures that the effectiveness of the interventions would not be diluted with scaling up and that the interventions are worth scaling up because they respond to the real needs and interests of patients and other stakeholders. In this sense, IKT can also be perceived as a necessary regulator of the upscaling process.

Fifth, as Milat et al [42] suggested, before scaling up an intervention, evidence of its effectiveness should ideally be provided through randomized controlled trials. In our case, it was impossible to manipulate exposure to the intervention, and so our evidence was from a natural experiment performed in the real world. Therefore, we skipped the randomized controlled trial step and went straight from our pilot project, a feasibility study, to the scale-up phase. However, the results of the pilot phase had already provided us information on scalability elements. Scaling up has been taking place, under different names, for several decades (especially in low- and middle-income countries for quickly stemming the spread of infectious diseases) [43], and current scale-up efforts in low- and middle-income countries show that scale-up strategies must be sufficiently flexible to respond to emerging questions [44]. Scaling up is still a new science and, as Milat et al [42] concede, must build flexibility in its application to real-world interventions.

Sixth, Quebec City, where our pilot took place, is almost unilingual and Francophone. Our workshops were scaled up to include libraries in Montreal, which has more immigrants and is more culturally diverse, and Westmount, which is more Anglophone. Although we did not measure these contextual differences in our sociodemographic questionnaires, the positive and consistent effect of citizen workshops on knowledge gain is a good indication that extending our model to more diverse populations will maintain acceptability and knowledge acquisition levels. However, this does not preclude the importance of adapting to different sociodemographic profiles when scaling up. Further adaptations may depend on the theme addressed, the target population, and the social situation. For example, the modalities of mass gatherings have changed dramatically during the COVID-19 pandemic. As a result, modifications in delivery will have to be made to our citizen workshops to follow public health recommendations.

Finally, we lacked the opportunity to conduct a complete economic (cost-effectiveness) analysis. However, our partial cost evaluation could be useful in the future for scaling-up studies, which so far have rarely included economic evaluations [43]. In addition, costs are considered an essential reporting item in the proposed guidelines for reporting on scaling-up studies [45]. Full economic evaluations in the real context of scaling up will also help choose efficient strategies involving the high-level engagement of patients and stakeholders across the scaling-up process and predicting the economic and human resource costs of further scale up.

### Limitations

The limitation of our study was, first, the fact that it had no comparison group. However, our earlier pilot project results helped us better understand some of the findings. It would be interesting to compare the costs of using an IKT approach to scale up our model without integrating patients and stakeholders, although it is ethically questionable. Second, we included only 1 patient caregiver in the research process. This could have limited the variety of patient perspectives in our research process. However, our patient caregiver, as the daughter of the patient, had not only a great life experience with the patient but also a substantial experience of health system use. Indeed, the caregiver supported her mother during different phases of her disease progression. Third, participants in the citizen workshops were self-selected citizens who responded to an advertisement for the workshop. However, self-selection sampling has some advantages: it reduces recruitment time, and self-selected participants are more likely to be committed to participate in the study (eg, more willing to spend time filling in the questionnaire) and to provide insights into the theme [46]. Nevertheless, we failed to meet the more vulnerable populations with lower literacy levels: half of the public in the workshops were university graduates and therefore not representative of Quebec's overall older adult population literacy level. Fourth, the data were collected using self-reporting tools; however, the impact of this on the effectiveness analysis should be, if anything, an underestimation of the knowledge gain among participants.

### Conclusions

This project successfully established a large-scale and successful KT bridge among researchers, clinicians, and citizens via public libraries. We found that scaling up a program of citizen workshops in public libraries resulted in high levels of knowledge gain, content appropriateness, and acceptability. The addition of an IKT approach involving patients and other stakeholders as research partners throughout the process and remunerating them improved the final product without harming the scale-up outcomes. These findings, based on citizen workshops integrating a computer-assisted presentation on scientific evidence and patient video clips plus a knowledge exchange session, highlight that an IKT approach and patient-oriented research should no longer be optional. This study provides a model for a dissemination practice that benefits the public by targeting and directly engaging them in the dissemination process. Public libraries are free and power-neutral educational institutions, and this simple and reproducible intervention is a ground-breaking knowledge translation model.

## Appendix

Multimedia Appendix 1 Evaluation forms.

Multimedia Appendix 2 Comparison of knowledge gain among participants in citizen workshops (using nonparametric tests).

## Abbreviations

IKT: integrated knowledge translation
KT: implementation science or knowledge translation
SPOR-SUPPORT: Strategy for Patient-Oriented Research Support for People and Patient-Oriented Research and Trials

## Data Availability

All data and materials used during the study will be available from the corresponding author (FL) upon request.
