# Supplementary material for: Scaling Up Citizen Workshops in Public Libraries to Disseminate and Discuss Primary Care Research Results: Quasi-Experimental Study
Source: JMIR Aging. 2022 Aug 19;5(3):e39016. doi: 10.2196/39016 (PMC9440407; doi:10.2196/39016)
Supplement: Multimedia Appendix 1 [file aging_v5i3e39016_app1.pdf]

UNITÉ DE SOUTIEN SRAP | QUÉBEC — Ville de Québec, Bibliothèque de Québec, Découvrir. Se divertir. Rêver. — Bibliothèques Montréal — Bibliothèque publique de Westmount / Westmount Public Library

Fiche d'évaluation de l'atelier « Des médicaments en trop? ».

Endroit (Bibliothèque) :

Date et heure (ex : Samedi 05 Novembre 2016 ; 11h)

À l'aide de l'échelle suivante, cochez la case qui correspond le mieux à votre opinion	**Pas du tout d'accord**	**Peu en accord**	**Plutôt d'accord**	**Tout à fait d'accord**
**Qualité et pertinence**				
1. Le contenu était adapté pour moi				
2. L'information présentée était claire				
3. Le contenu était pertinent				
4. La documentation remise vous semble utile pour le public				
**Structure de l'activité**				
5. L'atelier a permis d'atteindre les objectifs annoncés (voir objectifs)				
6. Le temps alloué aux différents points était suffisant				
7. J'ai pu participer de façon active				
**Animation**				
8. Les animateurs ont su créer une atmosphère propice à l'échange				
9. Il y avait une bonne complémentarité entre les animateurs				
10. Les animateurs ont répondu aux questions de manière claire et pratique				
**Dans l'ensemble**				
11. L'atelier a répondu à mes attentes				
12. Je recommanderais cette activité				

Identifiez deux caractéristiques que vous avez particulièrement appréciées dans cet atelier :

1. ______________________

2. ______________________

Écrivez une ou deux suggestions pour améliorer cet atelier

1. ______________________

2. ______________________

Sur une échelle de 10, à combien évalueriez-vous votre niveau de satisfaction globale vis-à-vis de <u>l'atelier « Des médicaments en trop?»</u>

…………../10

(Votre note)

Afin de vérifier si ce projet a contribué à augmenter votre niveau de connaissances sur certains aspects en lien avec votre pratique, svp, répondre aux questions suivantes :

Comment évalueriez-vous votre niveau de connaissance sur le thème abordé **avant** votre participation à ce projet?

0--------1--------2--------3--------4--------5--------6--------7--------8--------9--------10

Comment évalueriez-vous votre niveau de connaissance sur le thème abordé **après** votre participation à ce projet?

0--------1--------2--------3--------4--------5--------6--------7--------8--------9--------10

Autres sujets que vous aimeriez voir abordés par les médecins :

____

____

____

Autres commentaires et suggestions

____

____

____

Informez – vous des futures activités de « Ma santé, c'est mon choix » **Encerclez :** OUI NON

Courriel : ____________________________________

ENCERCLEZ : Je suis : UNE FEMME UN HOMME AUTRE

Quel âge avez-vous? ………….ans

ENCERCLEZ : Quelle est votre niveau d'éducation?
Primaire Secondaire Collégiale Universitaire

Merci d'avoir complété cette fiche d'évaluation.
