# Supplementary material for: Scaling Up Citizen Workshops in Public Libraries to Disseminate and Discuss Primary Care Research Results: Quasi-Experimental Study
Source: JMIR Aging. 2022 Aug 19;5(3):e39016. doi: 10.2196/39016 (PMC9440407; doi:10.2196/39016)
Supplement: Multimedia Appendix 2 [file aging_v5i3e39016_app2.pdf]

<u>Multimedia Appendix 3</u>: Comparison of knowledge gain among participants in citizen workshops (using non parametric tests) (N=276)[a].

**Characteristics**			**Population, n**	**Median knowledge gain (Q1, Q3)**	***P* value**[b]
**Participant characteristics**					
	**Sex**				0.49
		Male	208	2.00 (1.00, 3.00)	
		Female	68	2.00 (1.00, 3.00)	
	**Age** (years)		276		0.06
	**Highest educational level**				0.33
		Up to secondary	48	2.00 (1.00, 3.00)	
		College	70	2.00 (1.00, 3.00)	
		University	158	2.00 (1.00, 3.00)	
**Workshop characteristics**					
	**Time of day**				0.76
		Morning	72	2.00 (1.00, 3.00)	
		Afternoon	76	2.00 (1.00, 3.00)	
		Evening	128	2.00 (1.00, 3.00)	
	**Presence of the patient partner (PP)**				0.34
		Present	51	2.00 (1.00, 3.00)	
		Absent	225	2.00 (1.00, 3.00)	
	**Qualification of physician speaker**				0.43
		Physician +resident	18	1.00 (0.00, 3.00)	
		Resident only	19	2.00 (1.00, 3.00)	
		Physician only	239	2.00 (1.00, 3.00)	
**According to the physician speaker**			18 [c]	0.97[f]	
**According to the facilitator**			6 [d]	0.69[f]	
**According to the pair of moderators**			22 [e]	0.86[f]	

[a]N=276 (after deletion of observations with missing variables)

[b]P value of median test of knowledge gain of PIMs

[c]Number of physician's groups.

[d]Number of facilitator's groups.

[e]Number of pair of moderator's groups.

[f]*P* value of non-parametric ANOVA test.
